# Representing ECM composition and EMT pathways in gastric cancer using a new metastatic gene signature

**DOI:** 10.3389/fcell.2024.1481818

**Published:** 2024-11-05

**Authors:** Francesco Albano, Sabino Russi, Simona Laurino, Pellegrino Mazzone, Giuseppina Di Paola, Pietro Zoppoli, Elena Amendola, Chiara Balzamo, Ottavia Bartolo, Mario Ciuffi, Orazio Ignomirelli, Alessandro Sgambato, Rocco Galasso, Mario De Felice, Geppino Falco, Giovanni Calice

**Affiliations:** ^1^ Laboratory of Preclinical and Translational Research, Centro di Riferimento Oncologico della Basilicata (IRCCS-CROB), Rionero inVulture, Italy; ^2^ Laboratory of Stem Cell Biology, Department of Biology, University Federico II of Napoli, Napoli, Italy; ^3^ Laboratory of Stemness and Tissue Regeneration, Biogem S.c.a.r.l., Ariano Irpino, Italy; ^4^ Department of Molecular Medicine and Medical Biotechnologies, University Federico II, Napoli, Italy; ^5^ Istituto per l’Endocrinologia e l’Oncologia Sperimentale “Gaetano Salvatore” (IEOS), Consiglio Nazionale delle Ricerche (CNR), Napoli, Italy; ^6^ Endoscopy Unit, Centro di Riferimento Oncologico della Basilicata (IRCCS-CROB), Rionero inVulture, Italy; ^7^ Department of Translational Medicine and Surgery, Catholic University of Sacro Cuore, Roma, Italy; ^8^ Scientific Direction, Centro di Riferimento Oncologico della Basilicata (IRCCS-CROB) Via Padre Pio 1, Rionero inVulture, Italy

**Keywords:** gastric cancer, metastasis, transcriptomics and enrichment analysis, extracellular matrix, epithelial-to-mesenchymal transition, cell invasion

## Abstract

**Introduction:**

Gastric cancer (GC) is an aggressive and heterogeneous malignancy marked by cellular and molecular diversity. In GC, cancer cells invade locally in the stomach at stage I and can progress to metastasis in distant organs by stage IV, where it often becomes fatal.

**Methods:**

We analyzed gene expression profiles from 719 stage I and stage IV GC patients across seven public datasets, conducting functional enrichment analysis to identify a gene signature linked to disease progression. Additionally, we developed an *in vitro* model of a simplified extracellular matrix (ECM) for cell-based assays.

**Results:**

Our analysis identified a progression-associated gene signature (*APOD, COL1A2, FSTL1, GEM, LUM, and SPARC*) that characterizes stage IV GC. This signature is associated with ECM organization and epithelial-to-mesenchymal transition (EMT), both of which influence the tumor microenvironment by promoting cell invasion and triggering EMT.

**Discussion:**

This gene signature may help identify stage I GC patients at higher risk, offering potential utility in early-stage patient management. Furthermore, our experimental ECM model may serve as a platform for investigating molecular mechanisms underlying metastatic spread in gastric cancer.

## 1 Introduction

Gastric cancer (GC) is a neoplastic disease characterized by high intra- and intertumoral heterogeneity, which represents a great issue for the management of patients and may reduce the efficacy of current therapeutic strategies. Owing to this variability, several GC molecular subtypes have been defined (Fléjou, 2011; Ma et al., 2016; Oh et al., 2018; Sexton et al., 2020; Li and Wang, 2021; Russi et al., 2022) to improve the clinical management of patients. Currently, Lauren’s histological classification (Laurén, 1965) is still the main landmark for GC diagnosis, distinguishing two main histotypes of GC, the diffuse subtype (dGC) and the intestinal subtype (iGC), which are the most common. These histotypes have distinctive phenotypic, histological, and immunological features and, therefore, different prognoses (Laurén, 1965; Ma et al., 2016). As for other cancers, early diagnosis allows surgical resection, as the tumor is localized; however, as staging increases, survival drastically decreases: the 5-year survival rate is 65% for stage I patients, 35% for stage II patients, 25% for stage III patients, and only 20% of stage IV patients survive for more than 1 year (Smyth et al., 2020). Given this dramatic scenario, with our work, we aimed to dissect changes in gene expression during iGC progression from Stage I to Stage IV, highlighting core pathways and identifying new biomarkers driving this process. To this end, we merged several iGC gene expression databases to gain strong statistical power (Ooi et al., 2009; Cho et al., 2011; Lim et al., 2012; Lee et al., 2014; Oh et al., 2018; Subhash et al., 2018), finding that epithelial-to-mesenchymal transition (EMT) is one of the most relevant pathways controlling progression from Stages I to IV. Furthermore, we isolated a gene set (*FLORIO_NEOCORTEX_BASAL_RADIAL_GLIA_DN*) and a gene signature from it that was significantly upregulated in Stage IV vs*.* I. This intestinal gastric cancer progression signature (iGCPS) included genes encoding secreted proteins (apolipoprotein D - ApoD), ECM proteins (collagen 1 – Col1, follistatin-like 1 – Fstl1, lumican–Lum, secreted protein acidic and cysteine rich - Sparc), and a regulatory protein that participates in receptor-mediated signal transduction (GTP-binding protein overexpressed in skeletal muscle–Gem). From the iGCPS, we reconstructed a minimum model that approximates the iGC ECM composition likely influenced *in vivo* by the iGCPS, and we used this model to demonstrate its ability to promote *in vitro* cell invasion through EMT program activation.

## 2 Methods

### 2.1 Dataset composition

Workspace datasets GSE15460 (Ooi et al., 2009; Oh et al., 2018; Subhash et al., 2018), GSE13861 (Cho et al., 2011; Lim et al., 2012), GSE26253 (Lee et al., 2014; Oh et al., 2018), GSE26899 (Oh et al., 2018), GSE26901 (Oh et al., 2018) and GSE66229 (Oh et al., 2018) were downloaded from NCBI-GEO (https://www.ncbi.nlm.nih.gov/geo/); TCGA data were collected from https://www.cancer.gov/tcga as previously reported (Russi et al., 2019; Zoppoli et al., 2019) to obtain datasets comprising their respective info datasheet, including the annotation of the “*hystological_type*” parameter. First, their composition was executed by common row names identified by the symbol “Gene Symbol”. We subsequently used the ComBat method *sva* package (Leek and Storey, 2007; 2008; Leek et al., 2010) to apply batch effect correction. Finally, by using the *limma* package (Ritchie et al., 2015), we evaluated sample composition quality via principal component analysis (PCA). By this approach, we excluded 39 samples from the GSE15460 dataset since they significantly deviate from the other samples. Analysis of the clinical annotations revealed that the 39 excluded patients had an “age_at_surgery” value greater than that of other samples from the same dataset.

### 2.2 Dataset analyses

After dataset composition, we carried out differential gene expression analysis via the limma package, setting the statistical significance of the adjusted *p*-value <0.05. Survival analysis was performed via the common methods of the survival package (Borgan et al., 2001) (https://cran.r-project.org/web/packages/survival/), and we used the *survminer* package (https://cran.r-project.org/web/packages/survminer/) to obtain custom plots. Enrichment analysis was executed via the GSEA method of the *clusterProfiler* package (Yu et al., 2012) based on MSigDB gene set collection (Subramanian et al., 2005; Liberzon et al., 2011). Customized boxplots and customized enrichment plots were obtained via the *ggpubr* package (https://cran.r-project.org/web/packages/ggpubr/) and *Goplot* package (https://cran.r-project.org/web/packages/GOplot/), respectively; we created heatmaps via *pheatmap* (https://cran.r-project.org/web/packages/pheatmap/) and *ComplexHeatmap* packages (Gu et al., 2016). Finally, network visualization was implemented via the *Cytoscape* platform and its apps (https://cytoscape.org). All analyses were carried out in the R/Bioconductor environment (https://www.r-project.org/; https://bioconductor.org/) (Huber et al., 2015).

### 2.3 Cell culture, RNA extraction and real-time PCR

AGS cells and HEK293 T cells were purchased from ATCC (Manassas, United States) and grown, as previously described (Laurino et al., 2021), in DMEM-low glucose or DMEM (Thermo Fisher Scientific, Waltham, United States) supplemented with 10% heat-inactivated FBS, 2 mM L-glutamine, 100 U/mL penicillin, and 100 μg/mL streptomycin (Thermo Fisher Scientific, Waltham, United States), respectively. HEK293 T cells were used as packaging cells to produce lentiviral particles containing inducible human *GEM* coding sequences as previously described (Albano et al., 2018). Exogenous gene expression was controlled by adding 1 μg/mL doxycycline for 48 h before harvesting the cells for RNA extraction with TRIzol (Thermo Fisher Scientific, Waltham, United States), cDNA synthesis via a QuantiTect reverse transcription kit (Qiagen, Hilden, Germany) and real-time PCR via PowerUP Syber Green Master Mix (Thermo Fisher Scientific, Waltham, United States), as previously described (Vecchio et al., 2019; Rocca et al., 2020). The primers used for real-time experiments were as follows: Gem: FW 5′-TGA​ACG​AAC​CCT​GAT​GGT​TGA​TG-3’; RV 5′-CTC​GCT​CGG​TCT​GTG​ATT​GA-3’; βactin: FW 5′-GAG​CAC​AGA​GCC​TCG​CCT​TT-3’; and RV 5′-TCA​TCA​TCC​ATG​GTG​AGC​TGG-3’.

### 2.4 Collagen I invasion assay

Cell invasion was monitored via xCelligence Real Time Cell Analyzer (Agilent, Santa Clara, United States) equipped with a CIM-plate 16 as described by Knopfová et al. (2012) with some modifications. Briefly, for *Col1* conditions, 20 µL of 800 μg/mL Rat Tail Collagen I (Thermo Fisher Scientific, Waltham, United States) was used to coat the upper chamber of CIM-16 plates for 2 h at 37°C before the experiment. For *eCol1*, 1 μg/mL recombinant Sparc, follistatin 1, lumican (R&D Systems, Minneapolis, United States) and apolipoprotein D (Elabscience, Houston, United States) were added before rat tail collagen I polymerization. Next, 4 × 10^4^ AGS cells were plated in 8 wells in each of the upper chambers with serum-free media (plus 1 μg/mL doxycycline when required) or serum-free *eMedia* (plus 1 μg/mL doxycycline when required). Complete media was used as a chemoattractant in the lower chamber in 6 wells for each cell line, while serum-free media was used as a negative control in 2 wells for each cell line. Cell impedance changes were recorded every 15 min for 2 days. For quantification, the cell indices at the indicated time points were averaged from at least three independent measurements.

### 2.5 EMT evaluation by western blotting analysis

Under *Col1 conditions*, plates (60 mm) were coated with Rat Tail Collagen I solution diluted to 0.8 mg/mL following the manufacturer’s instructions; 2 mL of this solution was dispensed into each plate, which was allowed to polymerize for 2 h at 37°C. The *eCol1* matrix was prepared following the same procedure but with the addition of 1 μg/mL recombinant Sparc, Follistatin 1, Lumican and Apolipoprotein D before rat tail collagen I polymerization. *eMedia* was prepared by adding 1 μg/mL recombinant Sparc, Follistatin 1, lumican or Apolipoprotein D to the cell culture media. A total of 1 × 10^6^ AGS cells were seeded on Rat Tail Collagen I-coated plates and allowed to settle for 2 h before treatment and doxycycline addition. At the indicated timepoints, the media was removed, and 1.5 mL of 200 U/mL collagenase IV (Thermo Fisher Scientific, Waltham, United States) was added for 40 min at 37°C to digest the matrix and release the cells for downstream molecular analysis. Whole-cell lysates and Western blotting were performed as previously reported (Fiume et al., 2016).

### 2.6 EMT evaluation by confocal microscopy analysis

Individual wells of each 8-well cell culture slide (SPL Lifesciences, Pocheon, South Korea) were coated with 100 µL of *Col1* or *eCol1* as indicated for the cell invasion experiments. After collagen polymerization, 4 × 10^4^ cells cultured with standard media or *eMedia* with or without doxycycline (as described above) were seeded into each well and allowed to adhere overnight under the same conditions. The cells were subsequently fixed for 30 min at RT with 4% paraformaldehyde (pH 7.4), washed three times with PBS, and then permeabilized for 10 min with 0.1% Triton X-100 in PBS. Blocking was performed by using a 0.5% solution of BSA in PBS at RT for 30 min. The cells were then incubated overnight at 4°C with a rabbit anti-human β-catenin primary antibody (1:100, #8480, Cell Signaling Technology) and a mouse anti-human GEM primary antibody (1:50, sc-166891, Santa Cruz Biotechnology). After three washes with PBS, the cells were incubated with an Alexa Fluor 488-conjugated goat anti-rabbit IgG secondary antibody (A11070, Thermo Fisher Scientific, Waltham, United States) and an Alexa Fluor 647-conjugated goat anti-mouse IgG secondary antibody (A21236, Thermo Fisher Scientific, Waltham, United States) for 1 h at RT. The slides were washed three times with PBS and mounted with ProLong™ Glass Antifade Mountant with NucBlue™ (P36981, Thermo Fisher Scientific, Waltham, United States). Imaging was performed by using a Leica SP8 confocal microscope (Leica Microsystems) with a ×40 oil immersion objective.

## 3 Results

### 3.1 Differential gene expression analysis reveals a dysregulated molecular profile during iGC progression

**FIGURE 1:**
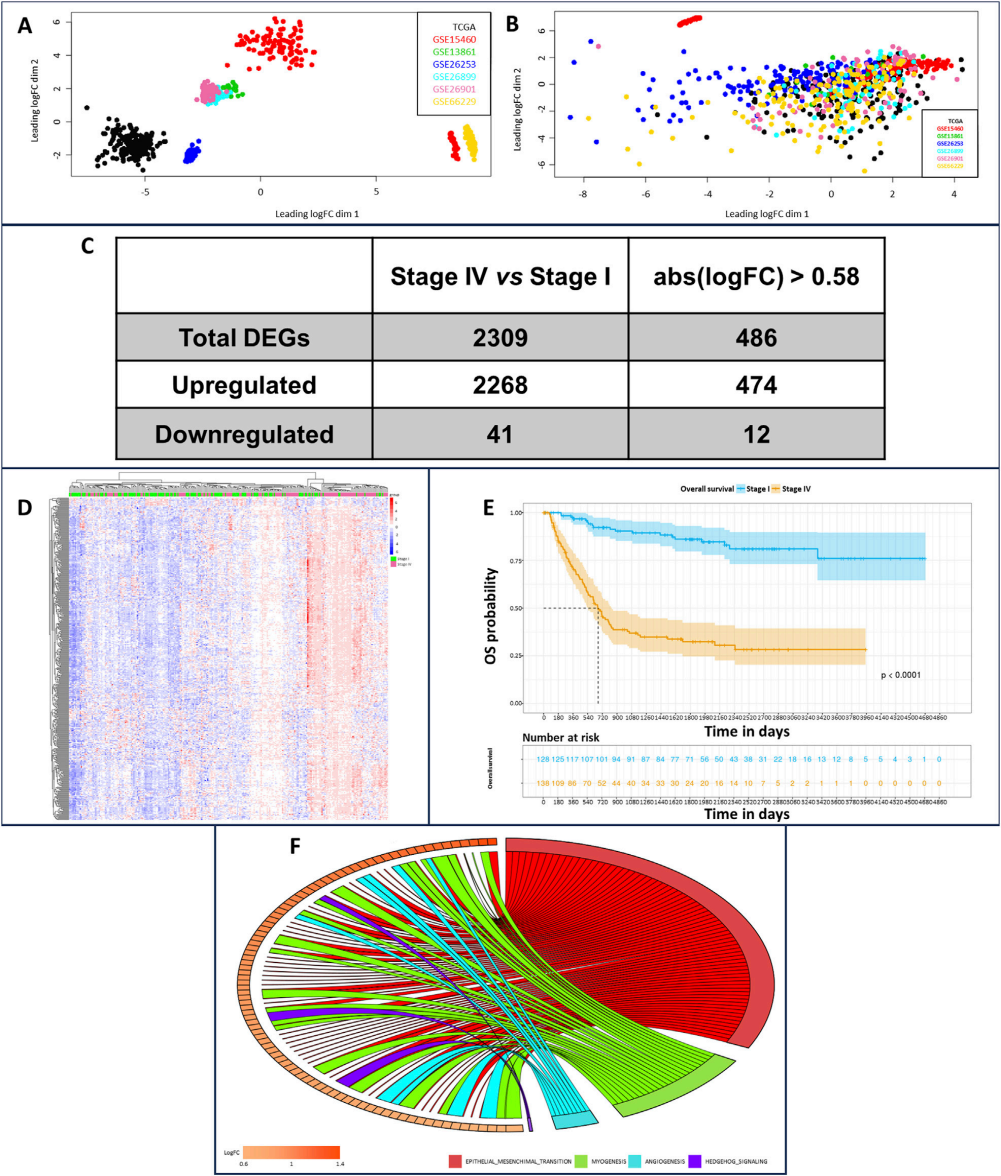
Principal component analysis of dataset sample distributions before **(A)** and **(B)** and after batch effect correction. In the upper part of plot B, in red, it is possible to observe the outlier samples characterized by higher “age_to_surgery” parameters; therefore, these samples were excluded from further analysis. Transcriptomic and survival differences between Stage IV and Stage I GC samples **(C)** Number of DEGs in stage IV vs*.* stage I patients, unfiltered and considering abs (logFC) > 0.58 as the cutoff. **(D)** Heatmap showing DEGs clustered according to GC pathologic stage (stage I patients in green, stage IV patients in pink, downregulated genes in blue, and upregulated genes in red). **(E)** OS analysis of stage I (blue samples) and stage IV patients (yellow samples) **(F)** Enrichment analysis relying on the hallmark MsigDB collections, comparing stage IV vs*.* stage I GC samples. It is possible to identify epithelial–mesenchymal transition as the most interesting enrichment, owing to the large number of gene connections.

We implemented a robust workspace of gene expression profiling data and their respective associated clinical information. This workspace was composed of 719 iGC samples generated by assembling seven different datasets. We applied batch effect correction to improve dataset consistency since principal component analysis (PCA) revealed marked heterogeneity (Figure 1A). Despite this correction, a very small part of the GSE15460 dataset, composed of 39 samples, defined a separate cluster (Figure 1B) characterized by an increased “*age_at_surgery*” parameter; therefore, we decided to exclude them from further analyses. Once the workspace was defined, we performed differential gene expression profiling analysis of Stage IV and Stage I iGC samples. We identified 2,309 significantly differentially expressed genes (DEGs) in stage IV vs*.* stage I patients (adjusted *p*-value < 0.05) (Figure 1C). Notably, 2,268 DEGs were upregulated (98%), and only 41 were downregulated, indicating that gradual upregulation of gene expression occurred during the progression of iGC from Stage I to Stage IV. We set the gene expression threshold value at abs (logFC) > 0.58 and found that 486 DEGs clustered according to iGC stages (Figure 1D). Overall survival (OS) analysis of our cohort revealed a distribution that was consistent with the literature. As expected, OS worsened during iGC progression (log rank test *p*-value <0.05) (Figure 1E). Concluding the overall preliminary analysis, we also highlighted the less variable DEGs by estimating the nonparametric coefficient of variation (npcv) (Gatto and Lilley, 2012) of all 486 Stage IV DEGs, both in the Stage I (n = 130) and in the Stage IV samples (n = 138). This could be helpful for identifying genes relevant to cancer cell survival and, consequently, for GC progression, as their expression remains consistent across samples. We list the top 5 LLVGs for each stage in Table 1 (A for Stages I and B for Stage IV).

**TABLE 1:** Less Level Variable Genes (LLVGs) for Stage I (A) and IV (B) patients.

Stage I	CV	Log FC
RPS24	0.1157	0.59
PCDHB5	0.1168	0.74
WWF	0.1176	0.76
NOV	0.1225	0.67
ARHGEF17	0.1233	0.73

Stage IV	CV	log FC
PFDN5	0.0795	0.74
RPS24	0.0847	0.59
TAF7	0.0848	0.65
MFGE8	0.0861	0.81

### 3.2 Functional enrichment analysis reveals biological categories associated with iGC progression

As our primary focus was to identify key regulators of iGC progression, we performed enrichment analysis on the differential gene lists, relying on the *canonical pathways, gene ontologies and hallmark* gene sets included in the MSigDB collection. For canonical pathways and gene ontologies, 458 and 277 significant categories (adjusted *p*-value <0.05) were enriched in Stage IV vs*.* I, respectively. Furthermore, 8 significantly enriched *hallmarks* (Figure 1F) were identified, all of which were overrepresented in Stage IV vs*.* I. Ranking the *hallmarks* using the normalized enrichment score (NES), we identified *EPITHELIAL_MESENCHYMAL_TRANSITION*, *ANGIOGENESIS*, *MYOGENESIS* and *HEDGEHOG_SIGNALING* as the top enriched hallmarks (Figure 1F; Supplementary Table S1). Notably, those categories had very high gene ratio scores, meaning that almost all the genes composing the hallmark gene set contributed to the enrichment being upregulated in Stage IV vs*.* Stage I (Supplementary Figure S1). Next, we analyzed all the MSigDB categories to determine which of them reverted their activation status from Stage I to Stage IV. The only one we could isolate was the *FLORIO_NEOCORTEX_BASAL_RADIAL_GLIA_DN* pathway. This pathway was present in the MSigDB collection CGP/C2 - chemical and genetic perturbations and canonical pathways and was overrepresented in Stage IV vs*.* I (0.55 enrichment score) and underrepresented in Stage II vs*.* I (−0.52 enrichment score). The core enrichment of this pathway was composed of 24 genes (Table 2), and considering genes upregulated in Stage IV vs*.* I with a logFC >1, we generated a gene signature of iGC stage progression (intestinal-type gastric cancer progression signature - iGCPS), composed of A*POD*, *COL1A2*, *GEM*, *FSTL1*, *LUM* and *SPARC*.

**TABLE 2:** Florio_neocortex_basal_radial_glia_dn core enrichment genes.

Gene	Log FC	*p*-value	Adj. *p*-value
APOD	1.26	6.08^–08^	8.32^–06^
SPARC	1.18	1.87^–08^	3.90^–06^
GEM	1.18	5.60^–11^	5.66^–08^
COL1A2	1.16	1.55^–07^	1.63^–05^
FSTL1	1.08	1.23^–08^	2.90^–06^
LUM	1.07	3.61^–07^	3.03^–05^
TUBB6	0.95	3.57^–08^	6.06^–06^
MFAP4	0.83	3.65^–06^	1.58^–04^
SERPINE 1	0.81	7.30^–09^	2.09^–06^
VCAM1	0.75	2.63^–06^	1.28^–04^
PRELP	0.69	7.88^–05^	1.36^–03^
TMEM47	0.60	1.03^–04^	1.65^–03^
EDNRB	0.55	1.60^–04^	2.28^–03^
TFPI	0.53	9.87^–04^	8.70^–03^
ITM2A	0.52	2.16^–03^	1.54^–02^
PDGFD	0.49	6.99^–04^	6.73^–03^
EMP1	0.46	6.52^–05^	1.19^–03^
SERTAD4	0.39	3.85^–03^	2.34^–02^
MCC	0.39	1.01^–03^	8.82^–03^
TRIM59	0.38	3.27^–03^	2.09^–02^
ELMO1	0.37	3.08^–03^	2.00^–02^
TGIF1	0.36	3.04^–03^	1.99^–02^
NID	0.26	4.25^–03^	2.51^–02^

### 3.3 The iGCPS expression level is correlated with gastric cancer stage

**FIGURE 2:**
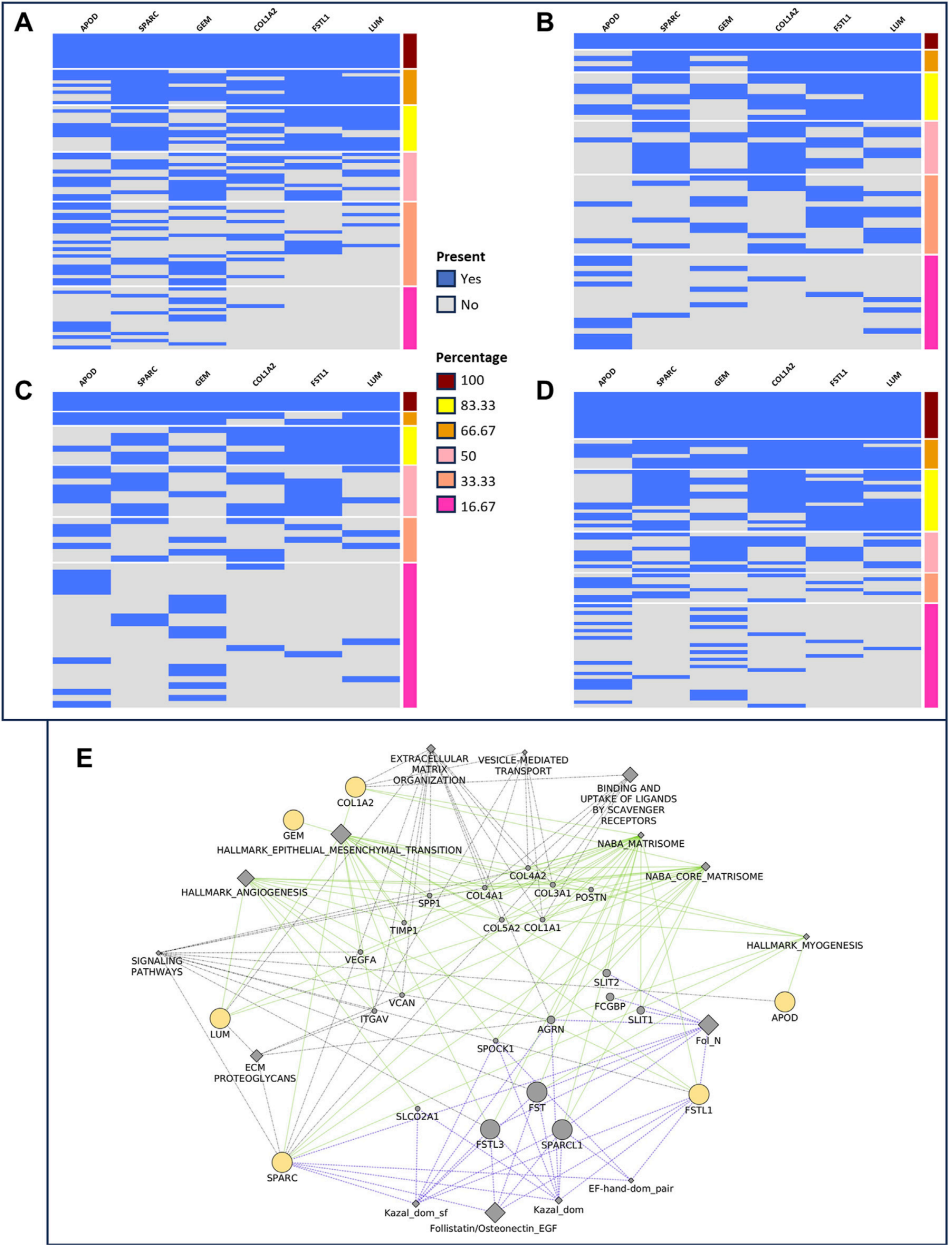
Grouping of stage I **(A, C)** and stage IV **(B, D)** patients according to the presence (blue) or absence (gray) of the genes composing the Florio signature. The gene expression level is divided into four quartiles, and extremes (1st - **(A, B)** and 4th - **(C, D)** are used to group samples. The number of patients with a 1st quartile expression level decreased from stage I **(A)** to IV **(B)**. Conversely, the number of patients in the 4th quartile of gene expression increased from stage I **(C)** to stage IV **(D)**. Visualization **(E)** of the network connecting iGCPS genes. Most of the categories able to connect genes, such as EMT, myogenesis and angiogenesis, match those identified through gene set enrichment analysis.

We used the iGCPS to group patients with Stages I and IV, considering the relative gene expression of the iGCPS in Stages I and IV. We considered the presence or absence of all the genes of the signature or the presence/absence of their permutations within the groups of patients. We then divided the gene expression levels into 2 groups: 1st quartile - low expression (gene expression level <1st quartile) and 4th quartile - high expression (gene expression level >3^rd^ quartile). We found the most interesting results when considering the presence/absence of the complete iGCPS. We observed that 10 out of 89 Stage I patients (11%, Figure 2A) expressed the iGCPS in the 1st quartile, whereas 3 out of 59 Stage IV patients (5%, Figure 2B) expressed the iGCPS in the 1st quartile. Interestingly, 3 out of 49 Stage I patients (6%, Figure 2C) expressed the iGCPS in the 4th quartile, whereas 13 out of 86 Stage IV patients (15%, Figure 2D) expressed the iGCPS in the 4th quartile.

We identified a new gene signature that could be helpful for managing GC patients. Its expression level is related to GC stage progression, and consequently, it could be related to OS and poor prognosis when strongly expressed in stage I patients.

### 3.4 Functional characterization of the iGCPS revealed its involvement in ECM organization and EMT

Given the clinical relevance of the iGCPS, we reconstructed the molecular and functional connections linking its genes. Notably, iGCPS genes encode a secreted protein (apolipoprotein D - ApoD) (Zhou and Luo, 2020; Takaya et al., 2023), ECM proteins (collagen 1 – Col1, follistatin-like 1 – Fstl1, lumican–Lum, secreted protein acidic and cysteine rich - Sparc) (Rossert and Decrombruggle, 2002; Barker et al., 2005; Lee et al., 2009; Chen et al., 2017; Mohammadzadeh et al., 2019; Li et al., 2021; Shan et al., 2021; Wu et al., 2021), and regulatory proteins that participate in receptor-mediated signal transduction (GTP-binding protein overexpressed in skeletal muscle–Gem) (Ward and Kelly, 2006).

We pinpointed a network to visualize connections between iGCPS genes via a) hallmark collection gene sets (http://www.gsea-msigdb.org/gsea/msigdb/collections.jsp - interactions highlighted in green); b) *the reactome pathway* database (https://reactome.org/ - interactions highlighted in gray); and c) the *InterPro* database (https://www.ebi.ac.uk/interpro/ - interactions highlighted in light blue). As expected, connections were mostly related to ECM organization, signal transduction and EMT activation (Figure 2E). Furthermore, most of the connections among the genes overlapped with the categories we previously found during the enrichment analysis (Figure 1F). These results led us to hypothesize that the iGCPS could recapitulate some of the most important features that can be observed in Stage IV ECM and that it could be in part responsible for the highly metastatic phenotype of tumor cells in this stage.

### 3.5 iGCPS induces invasion of gastric tumor cells through the ECM

#### 3.5.1 Model description

**FIGURE 3:**
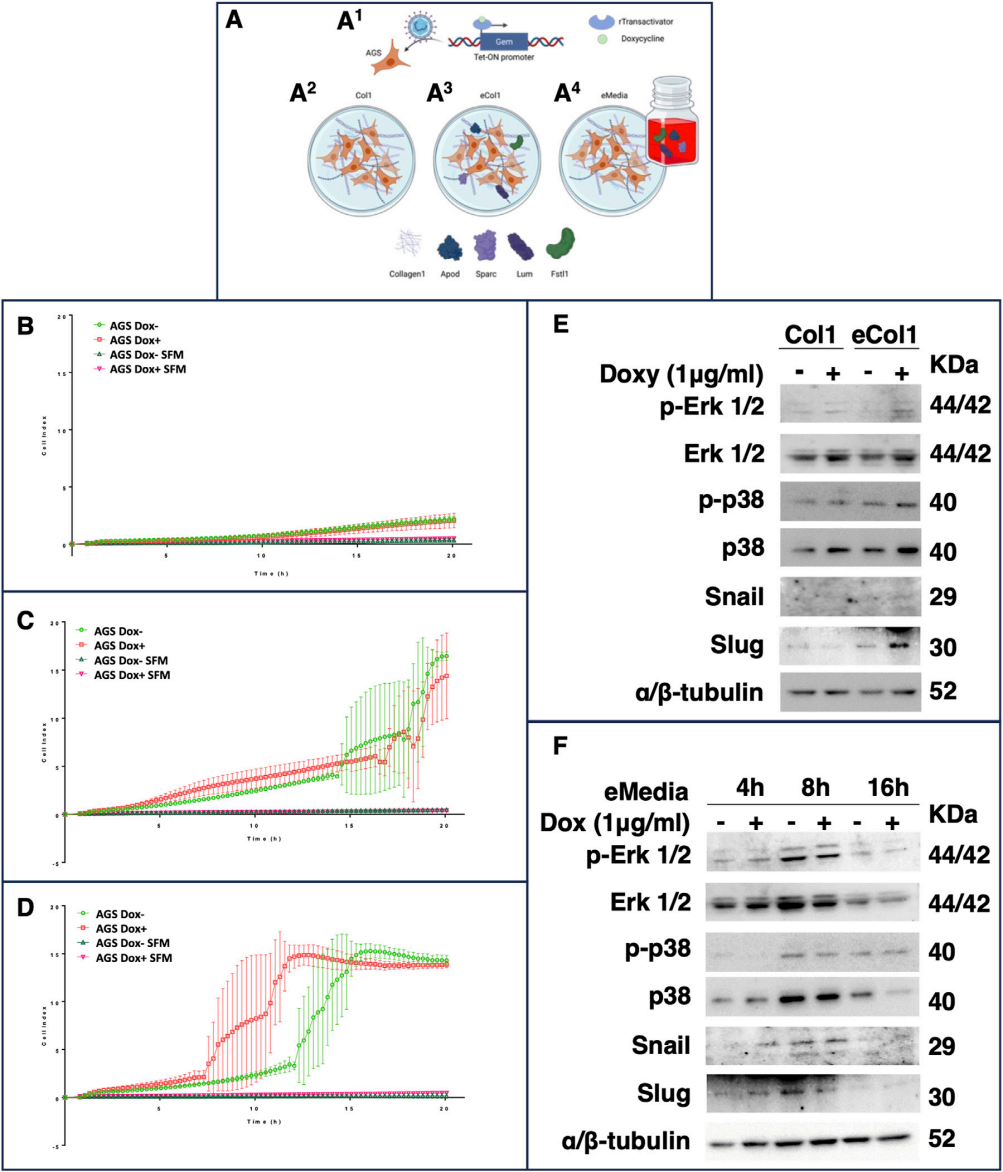
**(A)** Schematic representation of the experimental biological workflow. The gem CDS was cloned and inserted into an inducible lentiviral vector to generate a stable AGS cell line that expresses Gem in response to doxycycline treatment **(A1)**. The cells were then used for invasion experiments and for western blot and immunofluorescence analysis under different conditions: on a Rat Tail Collagen I matrix **(A2)**, on a Collagen I matrix enriched with the other iGCPS recombinant proteins **(A3)** or on a Collagen I matrix, and the other iGCPS recombinant proteins were used to enrich the cell culture media **(A4)**. Phenotypic analysis of AGS cells monitored for 24 h in real time via xCelligence and **(B)** grown on a rat tail collagen I matrix. The cells induced with or without doxycycline to produce Gem protein did not exhibit substantial cell invasion. The invasion of cells induced or not induced with doxycycline to produce Gem protein was repeated on collagen I enriched with the other iGCPS recombinant proteins **(C)** (eCol1). Under these conditions, massive invasion of AGS cells occurs a couple of hours after plating. Finally, the invasion of cells induced with or without doxycycline to produce Gem protein was monitored in real time through collagen I while the cells were treated with or without the other iGCPS recombinant proteins **(D)** (eMedia). Under these conditions, the invasion of noninduced AGS cells is similar to that measured under previous conditions (eCol1), while Gem production optimizes the process. Molecular analysis of the phenotypes shown in panels B and C **(E)** and panel D **(F)**. The expression and phosphorylation of the main MAPK responsible for cell movement, as well as the expression of the main TF regulators of EMT, were assayed at the indicated timepoints.

We decided to reproduce the iGCPS *in vitro* to verify whether and how this gene signature could influence cancer cell movement through the ECM, as the most evident phenotype of Stage IV GC is metastasis to distant organs. We attempted to shape some of the events that occurred during Stage I to IV GC progression. We chose the AGS cell line to model early-stage GC, as it represents one of the most widely used models of primary iGC; furthermore, this tumor cell line does not show strong metastatic potential during *in vitro* or *in vivo* experiments (Jin et al., 2015; Yang et al., 2015). First, we engineered AGS cells via lentiviral transduction to generate a stable and inducible cell line that expressed Gem only after doxycycline treatment (Figure 3A ^1^; Supplementary Figure S2). Next, we considered the upregulation of *COL1A2* (encoding the α2 chain of type I collagen) as a marker of increased collagen deposition in Stage IV. Type-I collagen is, in fact, the most abundant type of collagen in the human body and in the ECM (Rossert and Decrombruggle, 2002), and it is composed of two α1 chains and one α2 chain (Rossert and Decrombruggle, 2002). We therefore decided to grow AGS cells on a layer of Rat Tail Collagen I instead of growing them on a plastic surface to reproduce a very simplified ECM surrounding tumor cells (Figure 3A ^2^ - *Col1* conditions). Finally, to better understand the influence of the other secreted iGCPS proteins, we decided to embed them in the collagen 1 matrix (Figure 3A ^3^ - *eCol1* condition) or to use them as supplements of the cell culture medium (Figure 3A ^4^ - *eMedia* condition). We explored both cases, as we wondered whether iGCPS proteins could influence AGS cells via paracrine (*eCol1*) or exocrine (*eMedia*) mechanisms.

#### 3.5.2 Real-time monitoring of cell invasion

We performed invasion experiments via the xCelligence Real-Time Cell Analyzer (RTCA) DP instrument, which was equipped with a cellular invasion-migration plate (CIM-plate 16; a detailed description of the protocol is provided in the Materials and Methods). We adapted the experimental settings by substituting the Matrigel^®^ invasion matrix with Rat Tail Collagen I and continuously registering the AGS cell index (ci) for 20 h. First, we determined the basal invasion of AGS cells through Collagen I (*Col1* condition) by measuring similar Ci values between the AGS cells not induced (ci at 24 h, 2.18 ± 0.24; Figure 3B; green empty circles) or induced (ci at 24 h, 2.06 ± 0.65; Figure 3B; red empty squares), which is indicative of minimal, but not statistically significant, invasion of the cells with respect to the serum-free media (SFM) negative controls induced or not (0.49 ± 0.01 and 0.34 ± 0.16; Figure 3B; empty triangles; at 20 h, Col1 Dox-vs*.* Col1 SFM Dox-*t*-test, adj *p*-value 0.057; Col1 Dox + vs*.* Col1 SFM Dox + *t*-test, adj *p*-value 0.959; Col1 Dox-vs*.* Col1 Dox + *t*-test, adj *p*-value > 0.999). Adding the other members of the iGCPS (Apod, FSTL1, Lum and Sparc together as AFLS) to the experimental system drastically changed the scenario. The presence of AFLS proteins embedded in Collagen I (Figure 3C) allowed the cells to invade more efficiently at 2.5 h after plating, and the invasion of AGS cells significantly increased with respect to that of *Col1* cells (Figure 3C) (*t*-test adjusted *p*-value of 0.275), with a constant increase in ci (Figure 3C). Interestingly, Gem induction by doxycycline treatment did not influence further invasion, as induced (AGS Dox+, red empty squares) and uninduced cells (AGS Dox-, green empty circles) were comparable (Figure 3C). Finally, we used AFLS protein solubilized in serum-free cell culture media to treat AGS cells plated on collagen I (Figure 3D). In *eMedia* conditions, AGS cell invasion was not significantly different from that of *eCol1* cells until *15 h, whereas* AGS DOX-mediated cell invasion (Figure 3D) was greater than that of *eCol1* cells at the same timepoint (Figure 3D), considering both induced and not induced cells (*t*-test adjusted *p*-value of 0.010). The induction of Gem expression while simultaneously treating cells with AFLS proteins (Figure 3D - red empty squares) further increased the invasion of AGS cells compared with that of their noninduced counterparts (Figure 3D - green empty circles), with significantly greater cell invasion starting at 11 h (adjusted *p*-value of 0.008). Here, we demonstrated that the soluble form of the iGCPS protein can induce intracellular signaling that ultimately results in a highly invasive phenotype. Furthermore, Gem likely contributes to the optimization of this signaling pathway, resulting in increased aggressiveness of tumor cells.

### 3.6 iGCPS induces AGS cell invasion primarily by modulating p38 but not erk MAPK activation

A multitude of extracellular signals can induce mitogen-activated protein kinase (MAPK) signaling in eukaryotic cells, leading to broad intracellular responses to those stimuli, ranging from the regulation of metabolism to the control of the cell cycle and the regulation of cell motility (Cuadrado and Nebreda, 2010; Yang et al., 2015). Moreover, MAPK signaling can modulate EMT activation, initiating tumor metastasis to distant organs (Yang et al., 2015). We therefore explored the involvement of Erk1/2 MAPK and p38 MAPK in the mechanisms triggered by iGCPS and regulating the invasion of the AGS cells described above.

AGS cells were induced (or not) with doxycycline for 24 h before being detached from plastic dishes, seeded on a *Col1* 3D matrix and treated (or not) for an additional 24 h with doxycycline to maintain Gem expression before being harvested for Western blot analysis. Gem induction slightly increased Erk1/2 and p38 protein levels compared with those in noninduced cells (Figure 3E), even if their phosphorylation did not seem to be affected by Gem induction (Figure 3E). Next, the AGS cells were subjected to the same protocol but cultured on the *eCol1* 3D matrix. Compared with noninduced cells, growing *eCol1*-overexpressing cells did not lead to apparent changes in Erk1/2 expression or phosphorylation status (Figure 3E); similarly, Gem induction did not alter Erk1/2 expression or phosphorylation compared with that in noninduced cells. In contrast, growing cells on the *eCol1* matrix caused an increase in p38 protein expression and phosphorylation compared with those of *Col1* (Figure 3E), whereas Gem induction, with respect to noninduced cells, did not seem to strongly contribute. Finally, AGS cells were treated with *eMedia* containing soluble AFLS on a 3D collagen I matrix for an additional 4 h, 8 h or 16 h and doxycycline (to maintain Gem expression where requested). We chose these time points to match the timing of the invasion kinetics reported in Figure 3D (4 h, before the cells started to invade; 8 h, during cell invasion; and 16 h, when almost all the cells passed through the matrix to the lower chamber). *eMedia* treatment of noninduced cells from 4 h to 8 h had a marginal effect on only the Erk1/2 protein and its phosphorylation, as well as Gem induction, with respect to noninduced cells observed at the same time point. However, after 16 h of *eMedia* treatment, we observed a reduction in both Erk1/2 and its phosphorylation, even if we observed a weak contribution of Gem to the signaling pathway (Figure 3F). As previously observed for *eCol1* with respect to *Col1*, the strongest effect of exposure to AFLS was measured on p38 levels and p38 phosphorylation. During 8 h of *eMedia* treatment, p38 expression increased with respect to that at 4 h before returning to baseline after 16 h of treatment (Figure 3F). Interestingly, Gem induction was able to reduce p38 protein expression at 16 h, resulting in a net increase in the level of phosphorylated protein compared with that in noninduced cells and other time points (Figure 3F). We could conclude that iGCPS can induce cell invasion by activating p38 signaling.

### 3.7 iGCPS induces AGS cell invasion by activating the EMT program

**FIGURE 4:**
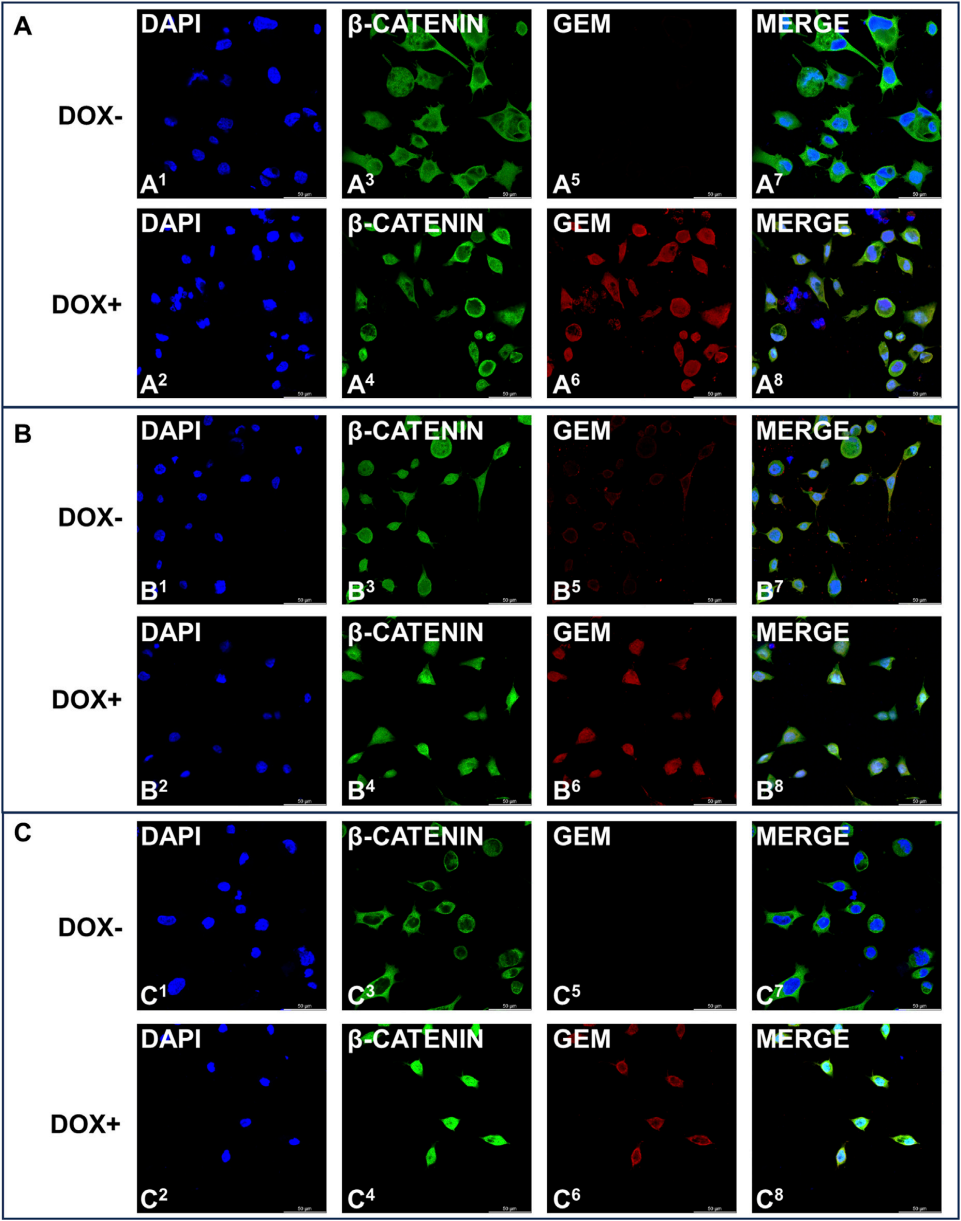
Immunofluorescence staining of Gem (red signals) and β-catenin (green signals) expression in AGS cells induced (even numbers) or not induced (odd numbers) with 1 μg/mL doxycycline and grown under **(A)** Col1 conditions **(B)** eCol1 conditions and **(C)** eMedia conditions. Nuclei were stained with DAPI (blue signal).

Activation of the EMT program is known to regulate the initiation of the metastatic process (Yeung and Yang, 2017), and Snail and Slug are among the main transcription factors that strongly drive EMT (Medici et al., 2008). β-Catenin is usually retained in the cytosol before being targeted for proteasomal degradation by ubiquitination, but extracellular stimuli and Wnt signaling can induce its nuclear accumulation and the activation of the EMT program (Basu et al., 2018). We explored whether iGCPS-driven AGS invasion could also be connected to Snail and Slug expression and β-catenin subcellular localization, as they can be considered molecular hallmarks of EMT process activation. Snail expression was not detected in the cells growing on *Col1,* and it was not affected by Gem overexpression (Figure 3E). On the other hand, Slug was expressed basally, but Gem expression induced a reduction in the expression of this transcription factor (Figure 3E). Finally, confocal microscopy analysis of intracellular β-catenin localization revealed that the protein was retained in the cytoplasm when AGS cells were grown on the *Col1* matrix and that its localization did not change following Gem induction with doxycycline (Figure 4A). Collectively, the molecular scenario depicted here is coherent with the results observed during invasion experiments (Figure 3B), as no EMT activation can be detected in the absence of all the members of the iGCPS. Snails were absent from both the *Col1* and *eCol1* culture scaffolds regardless of Gem induction (Figure 3E). On the other hand, Slug was induced in AGS cells on the *eCol1* matrix compared with that in cells on the *Col1* matrix, with Gem further contributing to the increase (Figure 3E). Finally, confocal microscopy analysis of intracellular β-catenin localization revealed that the protein can be detected both in the cytoplasm and in the nucleus of AGS cells grown on the *eCol1* matrix, with its localization not being further affected by Gem induction with doxycycline (Figure 4B). Collectively, these results are consistent with the phenotype observed during cell invasion experiments, as molecular analysis confirmed that iGCPS proteins embedded in the ECM favor the invasion of cells through the matrix by triggering Slug expression and β-catenin nuclear localization, even if Gem does not contribute to signaling. Finally, we analyzed EMT activation in the presence of *eMedia*. We detected the regulation of Snail and Slug expression with kinetics close to that of p38: increased expression at 8 h with respect to that at 4 h and decreased expression to baseline levels after 16 h (Figure 3F), with a limited contribution of Gem to this signaling. To evaluate β-catenin localization, we treated cells as described above and incubated them with *eMedia* for 16 h before they were fixed for confocal microscopy. Interestingly, we observed that eMedia treatment caused strong β-catenin nuclear localization only during Gem induction, whereas in noninduced cells, β-catenin localization was found to be mainly cytosolic (Figure 4C). Therefore, we can conclude that iGCPS is able to activate the EMT program, inducing cells to migrate through the matrix, and that Gem overexpression enhances Wnt/β-catenin signaling in tumor cells.

## 4 Discussion

Gastric cancer is a heterogeneous malignancy worldwide, with considerable intra- and interpatient variability that has led to several phenotypical and molecular classifications. One of the first GC classifications was published by Lauren in 1965 [3]. It is based on phenotypic and morphological parameters observed during histological analysis and divides GCs into well-differentiated (Intestinal type), poorly differentiated (diffuse type) and mixed types (Laurén, 1965). In this work, we focused on the intestinal GC subtype as defined by the Lauren classification, as it accounts for the highest number of cases with respect to other GC subtypes. Our aim was to characterize iGC progression in depth to improve the management of patient prognosis. We collected seven gene expression profile (GEP) datasets, which were selected based on accurate annotations. Following the data collection, we generated a comprehensive gene expression disease molecular workspace supported by the matched clinical data of 719 iGC patients. Gene expression profiling analyses highlighted several deregulated genes during iGC progression from the early to late stages. Furthermore, the upregulation was more noticeable in the advanced stage of GC classification, with a progressive increase in the number of DEGs, probably due to progressive uncontrolled cell proliferation that culminated with the spread of the cells throughout the body. This molecular evolution appeared even clearer when enriched hallmarks were overrepresented in stage IV than in stage I. EMT, in fact, was ranked as the top hallmark, as stage IV cells invade the stomach and metastasize to other organs. We also postulated that among the upregulated genes, those whose expression was less variable among samples (less level variable genes–LLVGs) could be critical for tumor cell survival and proliferation. Notably, the genes that we found in stage IV with the lowest coefficient of variation were consistent with this hypothesis, as we found genes involved in multiple intracellular pathways (Table 1), such as protein folding (*PFDN5*), RNA translation (*RPS24*), transcriptional activation (*TAF7*), induction of angiogenesis (*MFGE8*) and glycogenic amino acid transport (*SLC38A2*). We also evaluated whether the expression of LLVGs could be correlated with OS, but we found discordant results. This could be explained by the fact that even if those genes were similarly required by almost all the tumor cells during GC progression, they were not dominant in the regulation of the gene networks, probably due to the activation of many redundant pathways in the tumor cells. When we searched for specific molecular determinants that could govern progression through stages, we surprisingly found that the *FLORIO_NEOCORTEX_BASAL_RADIAL_GLIA_DN* pathway was the only pathway overrepresented in stage IV with respect to stage I and underrepresented in stage II with respect to stage I, indicating its specific activation in the late disease stage. This pathway was annotated for the first time by Florio et al. (2015) during transcriptomic characterization of the mouse and human neocortex. This analysis included genes enriched for extracellular matrix GO terms in human apical radial glia with respect to basal radial glial cells. Furthermore, they reported that endogenous ECM deposition and the production of ECM receptors by human apical radial glial cells contributed to their enhanced proliferative potential with respect to mouse cells (Florio et al., 2015). We therefore hypothesized that the overrepresentation of the pathway could be related to iGC stage progression. We analyzed the core enrichment of this pathway, highlighting all genes with the greatest changes in gene expression (logFC >1) when we compared Stage IV with Stage I, and we found that they were *APOD, COL1A2, FSTL1, GEM, LUM,* and *SPARC*. As this signature was upregulated in Stage IV patients compared with Stage I intestinal-type GC patients, we referred to this signature as iGCPS, the intestinal-type gastric cancer progression signature. Interestingly, many of the genes of the iGCPS were previously related to several types of cancers, advanced stages, and the regulation of the ECM composition (Chen et al., 2017; Zhou and Luo, 2020; Shan et al., 2021; Wu et al., 2021). Notably, *GEM* encodes an intracellular GTP-binding protein that participates in receptor-mediated signal transduction (Ward and Kelly, 2006). It has been reported that Gem can bind to Rho kinase β (ROCKβ), changing the substrate specificity of ROCKβ rather than blocking its catalytic activity (Ward and Kelly, 2006). Interestingly, two different methods of cell movement through a 3D matrix have been described thus far, the mesenchymal and ameboid methods, which are interconvertible and antagonistic (Sahai and Marshall, 2003; Sanz-Moreno et al., 2008; Amano et al., 2010). Different requirements for Rho-ROCK signaling can drive cells toward one of the two phenotypes, inducing them to invade the tissue via EMT or the mesenchymal-to-ameboid transition (MAT) (Sahai and Marshall, 2003; Sanz-Moreno et al., 2008; Paňková et al., 2010), with Gem likely involved in regulating this switch of phenotypes. *APOD* codes for apolipoprotein D (ApoD), a secreted apolipoprotein constituting HDL particles that can also be detected in plasma blood (Takaya et al., 2023). Apod has a controversial role in cancer, mostly depending on where it is more abundant, in the tumor or in the stroma (Zhou and Luo, 2020). Indeed, one of the described effects of ApoD is to induce senescence in the stroma, thus generating an environment favorable for tumor cell invasion (Zhou and Luo, 2020). The genes *COL1A2, FSTL1, LUM,* and *SPARC* encode proteins that structurally compose the ECM and that are frequently altered in cancer, conferring a poor prognosis and invasive phenotypes (Barker et al., 2005; Lee et al., 2009; Shieh et al., 2011; Zhang et al., 2022). Once the iGCPS was identified, we used it to divide patients who presented the lowest gene expression levels (1st quartile) from patients with the highest gene expression levels (4th quartile). We noticed that the number of patients in the 1st quartile decreased from stage I to stage IV, whereas the number of patients in the 4th quartile increased from stage I to stage IV, suggesting that the signature could be used for the early identification of patients with aggressive disease. Given that the clinical potential of the iGCPS is clear, we explored the mechanism that could account for stage progression. We retrieved a network connecting the genes composing the iGCPS, and as expected, we obtained again that genes were linked together by EMT, ECM composition/organization, and activation of signaling pathways. This finding was not surprising, as in Stage IV iGC, metastatic progress is strongly active, causing cancer cells to spread throughout the body, and metastatic cells must remodel the ECM deeply to enter the bloodstream and colonize distant organs. We therefore decided to further explore the mechanisms and pathways regulating iGC progression, generating a novel iGC progression model based on the iGCPS. We used AGS cells as a tumor model, as those cells were established from the primary tumor of a patient diagnosed with iGC. Furthermore, AGSs are not efficient at invading the ECM or in developing xenografts in mice (Jin et al., 2015; Yang et al., 2015). We demonstrated that the presence of iGCPS in the ECM is sufficient to initiate the EMT process and to induce cells to remodel the ECM to invade it. In addition, we described that iGCPS members can more efficiently induce EMT and ECM invasion when they are available as soluble proteins, as they probably interact with plasma membrane receptors, triggering signaling pathways related to MAPK and Wnt/β-catenin, a mechanism that has already been reported for Fstl1 (Zhang et al., 2022) during kidney fibrosis. These discoveries are extremely relevant for the management of iGC. Indeed, the Tissue Protein Atlas (https://www.proteinatlas.org/) reports that iGCPS proteins can all be detected in human blood (except for Gem, which, as already mentioned, is not secreted by cells). Therefore, it could be possible to quantify their presence in human blood to screen for and diagnose iGC in patients before it can be fully developed, thus improving patient prognosis and survival. In addition, further studies must be conducted to identify and characterize plasma membrane receptors involved in iGCPS-mediated signal transduction, as that information could be relevant for the design and screening of drugs, small molecules or monoclonal antibodies that could interfere with those signaling pathways, hampering the initiation of metastatic spread. Finally, it could be interesting to screen a single cell sequencing dataset via the iGCPS to detect the candidate cell type that could primarily be responsible for the increased secretion of the iGCPS, as it could also be responsible for conditioning the TME, supporting cancer cell growth and transformation.

## 5 Conclusion

Taken together, our results highlighted key modifications occurring during iGC progression from stage I to IV through the composition and analysis of comprehensive disease data from GC patients. Furthermore, we discovered a strong prognostic signature that is able to predict the presence of an aggressive tumor in early-stage GC, biologically demonstrating the early activation of the EMT process that makes cells prone to ECM invasion and metastatic spread.

## Data Availability

All data generated or analyzed during this study are included in this published article, and its supplementary information files, and are available upon reasonable request to the corresponding authors. Publicly available datasets were analyzed in this study. This data can be found here: GSE15460 GSE13861 GSE26253 GSE26899 GSE26901 GSE66229 https://www.cancer.gov/tcga.
