# Supplementary material for: Representing ECM composition and EMT pathways in gastric cancer using a new metastatic gene signature
Source: Front Cell Dev Biol. 2024 Nov 5;12:1481818. doi: 10.3389/fcell.2024.1481818 (PMC11573575; doi:10.3389/fcell.2024.1481818)
Supplement: Supplementary file 1 [file Image2.PDF]

**Supplementary figure 2**

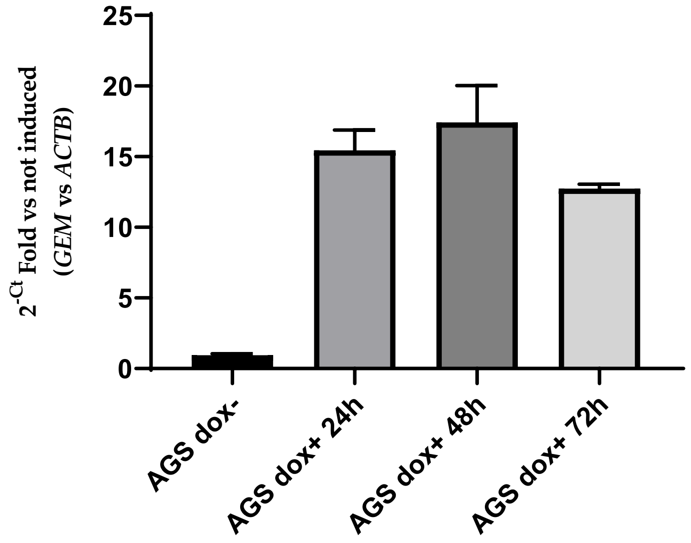


**Supplementary figure 2** Real-Time PCR evaluation of *GEM* expression, after generating transgenic AGS cells. Cells were induced with 1µg/ml Doxycycline for 24, 48 and 72h, or left not induced and then processed for RNA extraction and Real-Time PCR.
