# Supplementary material for: Representing ECM composition and EMT pathways in gastric cancer using a new metastatic gene signature
Source: Front Cell Dev Biol. 2024 Nov 5;12:1481818. doi: 10.3389/fcell.2024.1481818 (PMC11573575; doi:10.3389/fcell.2024.1481818)
Supplement: Supplementary file 3 [file Image1.PDF]

**Supplementary figure S1**

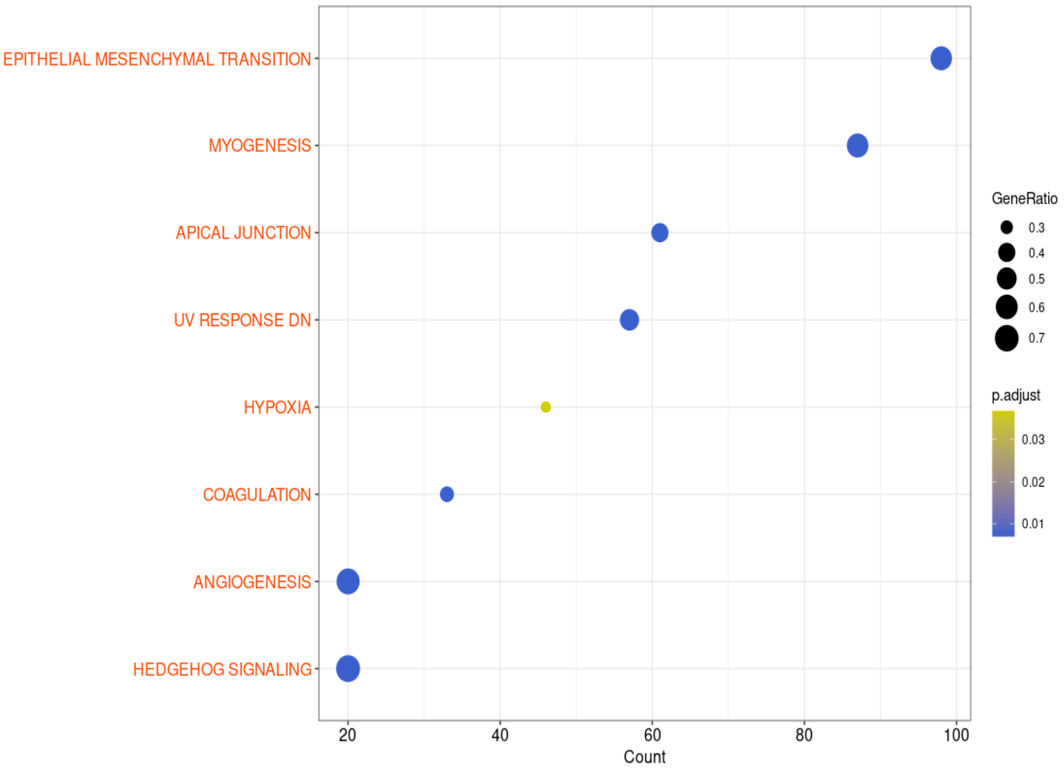


**Supplementary figure 1** Enrichment analysis relying on the Hallmark MsigDB collection, comparing stage IV *vs* stage I GC samples. The dot size indicates the gene ratio of each category, and it increase consistently with gene coverage of the respective category.
